# Cognitive Deficits, Apathy, and Hypersomnolence Represent the Core Brain Symptoms of Adult-Onset Myotonic Dystrophy Type 1

**DOI:** 10.3389/fneur.2021.700796

**Published:** 2021-07-01

**Authors:** Jacob N. Miller, Alison Kruger, David J. Moser, Laurie Gutmann, Ellen van der Plas, Timothy R. Koscik, Sarah A. Cumming, Darren G. Monckton, Peggy C. Nopoulos

**Affiliations:** ^1^Department of Psychiatry, Carver College of Medicine, University of Iowa, Iowa City, IA, United States; ^2^Department of Neurology, Indiana University School of Medicine, Indianapolis, IN, United States; ^3^Institute of Molecular, Cell and Systems Biology, College of Medical, Veterinary and Life Sciences, University of Glasgow, Glasgow, United Kingdom; ^4^Department of Neurology, Carver College of Medicine, University of Iowa, Iowa City, IA, United States; ^5^Department of Pediatrics, Carver College of Medicine, University of Iowa, Iowa City, IA, United States

**Keywords:** myotonic dystrophy, apathy, hypersomnolence, cognition, depression, fractional anisotropy

## Abstract

Myotonic dystrophy type 1 is the most common form of muscular dystrophy in adults, and is primarily characterized by muscle weakness and myotonia, yet some of the most disabling symptoms of the disease are cognitive and behavioral. Here we evaluated several of these non-motor symptoms from a cross-sectional time-point in one of the largest longitudinal studies to date, including full-scale intelligence quotient, depression, anxiety, apathy, sleep, and cerebral white matter fractional anisotropy in a group of 39 adult-onset myotonic dystrophy type 1 participants (27 female) compared to 79 unaffected control participants (46 female). We show that intelligence quotient was significantly associated with depression (*P* < 0.0001) and anxiety (*P* = 0.018), but not apathy (*P* < 0.058) or hypersomnolence (*P* = 0.266) in the DM1 group. When controlling for intelligence quotient, cerebral white matter fractional anisotropy was significantly associated with apathy (*P* = 0.042) and hypersomnolence (*P* = 0.034), but not depression (*P* = 0.679) or anxiety (*P* = 0.731) in the myotonic dystrophy type 1 group. Finally, we found that disease duration was significantly associated with apathy (*P* < 0.0001), hypersomnolence (*P* < 0.001), IQ (*P* = 0.038), and cerebral white matter fractional anisotropy (*P* < 0.001), but not depression (*P* = 0.271) or anxiety (*P* = 0.508). Our results support the hypothesis that cognitive deficits, hypersomnolence, and apathy, are due to the underlying neuropathology of myotonic dystrophy type 1, as measured by cerebral white matter fractional anisotropy and disease duration. Whereas elevated symptoms of depression and anxiety in myotonic dystrophy type 1 are secondary to the physical symptoms and the emotional stress of coping with a chronic and debilitating disease. Results from this work contribute to a better understanding of disease neuropathology and represent important therapeutic targets for clinical trials.

## Introduction

Myotonic dystrophy type 1 (DM1) is a trinucleotide repeat disorder, classically characterized with motor symptoms of prolonged muscle contractions (myotonia), progressive muscle wasting, and weakness (1). As a multisystemic disease, DM1 manifests with many additional non-motor symptoms including cataracts, heart conduction abnormalities and arrythmias, gastrointestinal abnormalities, endocrine abnormalities, insulin resistance and diabetes, and respiratory failure. Other non-motor symptoms, including cognitive deficits, sleep disturbances and affective symptoms, are thought to be due to CNS pathology in DM1, a feature of the disease that has had increasing focus over the past 5–10 years. White matter (WM) microstructure pathology is one of the most robust and reproducible observations in DM1, supported by several neuroimaging studies to date (2–13). Fractional Anisotropy (FA, obtained via diffusion tensor MR imaging [DTI]) provides a measure of WM microstructural integrity by quantifying local restrictions of the direction of diffusion of water molecules and ranges from 0 (e.g., cerebrospinal fluid) to ~1 (e.g., highly myelinated WM fiber bundles). In DM1, FA has been shown to be globally reduced, with limited regional specificity (3–14).

As a single gene disorder, there has been significant progress toward developing gene knock-down or other therapies directed at the core site of pathology in DM1. To treat the CNS associated symptoms of DM1, there is consideration and progress toward delivery of these therapies directly to the brain (15). In the context of developing endpoints for monitoring centrally administered therapy, it is crucial to distinguish what features of DM1 are truly related to brain pathology and which features may be secondary manifestations of living with a chronic neurologic disease. This will move the field toward more robust clinical targets, biomarkers, and endpoints for clinical trials. The symptoms of DM1 considered directly related to brain pathology include cognitive deficits, apathy, hypersomnolence/fatigue, and affective symptoms of depression and anxiety (15). These symptoms can have a significant burden on both the patient and their caregiver (15–17).

It is important to consider the potential relationships between the symptoms of DM1 when evaluating whether or not they represent core features of the disease. General cognitive deficits are severe in congenital and childhood forms of DM1 (18), but even in adult-onset DM1, we have shown that Full Scale IQ (FSIQ) is significantly lower than unaffected healthy adults (19, 20). Although verbal skills are typically less affected than visuospatial skills, a significantly lower FSIQ highlights a generalized deficit in cognitive skills. There is also significant evidence for global cognitive deficits in adult-onset DM1 present across several different domains (21). For patients navigating the burden of having a degenerative neurologic disorder, deficits in cognitive skills can result in sub-optimal coping strategies potentially driving secondary emotional problems, including depression and anxiety.

The majority of studies evaluating depression in DM1 have utilized rating scales such as the Beck Depression Inventory (BDI) (6, 9, 10, 14, 16, 22–28), the Hamilton Rating Scale for Depression (HAM-D) (29–34) or a variety of other standardized, self-rating scales (35–48), and comparison groups were often adults with no major medical illnesses. However, rating scales such as the BDI-II not only assess mood and cognitive symptoms such as sadness, suicidality, and guilt, but also somatic symptoms such as fatigue and sleep disturbance. When using depression rating scales, it is vital to determine whether the scores are increased due to higher somatic ratings or because of depressed mood, as many of the other symptoms of DM1 (hypersomnolence, fatigue) will contribute to the overall depression score.

To better understand whether these symptoms in DM1 are truly related to brain pathology, a few studies have evaluated the relationship between cerebral WM FA and symptoms. Several studies have shown a relationship between lower FA, lower cognitive function (9), and hypersomnolence (4). Yet, no study has systematically evaluated all of these brain symptom measures together, while accounting for relationships among measures. In addition, if a symptom of a progressive, worsening disease is truly related to the pathology of that disease, that symptom would worsen over the course of time. Therefore, the association of a symptom with disease duration would also support the notion that that symptom was due to progressive brain pathology, as shown previously with cognitive performance (49, 50).

The Iowa DM1 study evaluates brain structure and function in adult-onset DM1. Here, as part of this larger study, we evaluate measures of cognitive/behavioral symptoms, including FSIQ, depression, anxiety, apathy (obtained from both the participant and an informant, as patients often under-rate this symptom), and hypersomnolence. First, we evaluated the relationship between cognitive impairment to ratings of depression, anxiety, apathy, and hypersomnolence. Then, relationships between these clinical measures and cerebral FA were compared. Finally, disease duration (as a marker of disease progression) was used as a predictor for these measures and cerebral FA. Our results indicate that cognitive deficits, apathy, and hypersomnolence represent that core brain symptoms of DM1.

## Patients and Methods

### Participants

The Iowa DM1 Brain study targets individuals with adult-onset DM1 (diagnosis after the age of 18 years old). Participants were recruited from our multidisciplinary specialty clinic for DM1 at the University of Iowa and through the Myotonic Dystrophy Foundation. Healthy adults were recruited from spouses of DM1 participants and from the Iowa City area via advertisements. Exclusion criteria for all participants included: MRI contraindication, a history of serious head injury that resulted in a hospital stay, or a chronic neurological disorder other than DM1. Healthy adults were additionally required to be without history of substance abuse, psychiatric disease, or major medical disease, including heart disease, sleep disorder, vascular disease, uncontrolled hypertension, cancer, diabetes mellitus, lung disease, and autoimmune conditions. A total of 8 potential DM1 participants and 9 potential healthy control participants were either excluded or did not meet inclusion criteria for the study and were excluded. The current sample included 39 individuals with DM1 and 71 healthy adults. Demographics are displayed in Table 1. There was no significant difference in sex (χ^2^ = 0.068, *df* = 1, *p* = 0.794) or age [*t*_(108)_ = 0.96, *p* = 0.337] between groups. Disease duration was defined as the time between onset of the first motor symptom of DM1 and time of assessment. Mean disease duration for this group was 12.9 years with a range of 2.4 years to 28.9 years. As far as the severity of muscular impairment, the DM1 group had 13 individuals with a score of one (no impairment), 24 with a score of two (minimal signs), nine with a score of three (distal weakness), four with a score of four (mild to moderate proximal weakness), and none with a score of five (severe proximal weakness).

**Table 1:** Demographics of study sample.

			**Healthy controls**	**DM1**
Sample	n		71	39
Sex	n	Males	25	12
		Females	46	27
Age at evaluation	Mean (SD)		43.22 (13.07)	45.49 (9.03)
Age at disease onset	Mean (SD)		n/a	32.35 (9.59)
Disease Duration	Mean (SD)		n/a	12.87 (7.38)
MIRS	n	MIRS 1	-	13
		MIRS 2	-	24
		MIRS 3	-	9
		MIRS 4	-	4
		MIRS 5	-	0
CTG	Range		5–43	81–501
	Median		13	146

All participants gave written, informed consent prior to enrolling in the protocol in accordance with the Declaration of Helsinki. The study was approved by the University of Iowa Institutional Review Board.

Genotyping of CTG repeat in DM1-affected participants was completed by small-pool PCR (SP-PCR) (51). For each patient, four reactions were completed, each using 300 pg genomic DNA template derived from blood leukocytes. CTG repeat lengths were estimated by comparison against DNA fragments of known length and molecular weight markers, using CLIQS software (TotalLabs UK Ltd.). The lower boundary of the expanded molecules in SP-PCR was used to estimate the progenitor (inherited) allele length (ePAL) (52). The mean ePAL for the DM1 group was 146 with a minimum of 81 and a maximum of 501. Repeat lengths for the non-disease-causing alleles from all participants were estimated using the Illumina MiSeq platform, broadly as described for Huntington disease alleles (53). Primers that flanked the CTG repeats were used to amplify across the repeat region, also adding barcoded sequencing adapters to generate the sequencing library. The resulting reads were aligned against reference sequences comprising the 5′ and 3′-flanking sequences separated by 0–100 CTG repeats. The genotype was the CTG repeat length to which the highest number of sequence reads were mapped.

Research staff, clinicians, and scientists involved in this study remained blind to the participant's clinical condition (CTG expansion length and muscular impairment). However, this was not always possible when participants exhibited moderate-to-severe symptoms of DM1 during the study. All clinical scales and measures were administered by a trained examiner experienced in DM1. All data were de-identified and all participants consented to non-disclosure of genetic results obtained as part of the study.

### Motor Testing

Severity of muscle weakness was measured using the Muscle Impairment Rating Scale (MIRS) during examination by a neuromuscular specialist experienced in DM1, blinded to the participants' genetic status (54). This scale evaluates muscular impairment severity according to an ordinal 5-point scale as follows: (1) no muscular impairment, (2) minimal signs, (3) distal weakness, (4) mild to moderate proximal weakness, and (5) severe proximal weakness.

### General Cognitive Abilities

Participants completed the Wechsler Adult Intelligence Scale-IV (WAIS-IV) to estimate Full Scale IQ (55).

### Depression and Anxiety

The Beck Depression Inventory (BDI-II) is a widely used questionnaire measuring self-reported symptoms of depression on a 4-point Likert scale ranging from 0 to 3 (56). A total score is summed and can be interpreted clinically as 0–13 being minimal depression; 14–19 being mild depression; 20–28 being moderate depression; and 29–63 being severe depression. The Beck Anxiety Inventory (BAI) is a corollary self-report questionnaire (21 questions on a 4-point Likert scale) assessing symptoms related to anxiety.

### Sleep Quality

The SCOPA-Sleep (Scales for Outcomes in Parkinson's Disease-Sleep) survey was used to assess daytime sleepiness (57). The self-report scale includes six items in subscale D: Sleeping during the day and evening, which were summed to calculate hypersomnolence scores. Items used to measure overall sleep quality from subscales A: Use of sleeping tablets, B: Sleeping at night, and C: Global evaluation of sleeping at night, were not included in the analysis.

### Apathy

The Apathy Evaluation Scale (AES) was used to determine self-reported degree of apathy (58). The AES includes 18 items that are rated on a 4-point Likert scale. Items were summed to create a total score, where higher scores represent increased apathy. In addition, we had a subset of 24 of the 39 participants with DM1 who had an informant fill out the informant version of the scale.

### Magnetic Resonance Imaging

Individuals who participated before June 2016 (49 controls, 25 DM1) were scanned using a 3T Siemens TrioTIM scanner (Siemens AG, Munich, Germany; 12 channel head coil, software version: syngo B17). Those who participated after June 2016 were scanned using a 3T General Electric Discovery MR750w scanner (GE Medical Systems, Chicago, Il, 16 channel head and neck coil, software versions: 25.0, 25.1, and 26.0) (21 controls, and 13 DM1). Participants completed DWI acquisitions with either a single-shell (B1000, 64 directions), multi-shell (B1000 and B2000, 29-30 directions per shell), or both. Diffusion-weighted images were collected using echo planar recovery magnitude sequences collected in the axial plane. Anatomical T1-weighted and T2-weighted images were collected and used for co-registration, normalization, and labeling purposes using acquisition parameters described previously (59).

### Fractional Anisotropy

Diffusion-weighted images were processed using standard procedures of the FMRIB Diffusion toolbox from the FSL software package (http://www.fmrib.ox.ac.uk/fsl), where phase encoding distortion and eddy current artifacts were removed using topup and eddy tools respectively (60, 61). Following correction, diffusion tensor models were generated using dtifit, and from these tensors, scalar measures of anisotropy (FA) were calculated. B0 maps were co-registered to T2-weighted image for each participant, which were in turn registered to their T1-weighted images, which were normalized to a standard space. All registrations consisted of rigid, affine, and non-linear (symmetric normalization) components and were conducted using Advanced Normalization Tools (62) and were applied together in a single interpolation step to avoid compounding interpolation errors. We focused on cerebral white matter FA for the current analysis, rather than regional WM FA, given prior research indicating lack of regional specificity of WM FA deficits in DM1.

### Statistical Analyses

All statistical analyses were performed using R (version 3.6.2). Linear regression models were run to compare group differences across groups in depression, anxiety, apathy, sleep, FSIQ and cerebral WM FA. All models were controlled for age, sex and a sex-by-group interaction. The semi-partial coefficient of determination (R^2^) for the model, and 95% confidence intervals were calculated. Effect sizes were considered very small (R^2^ < 0.1), small (0.1 < R^2^ < 0.3), moderate (0.3 < R^2^ < 0.5), and large (0.5 < R^2^) (63). The interaction term was removed from the model if not significant at *p* < 0.05 and removed from further analyses.

A second set of models determined the effect of FSIQ (predictor variable) on the clinical measures of depression, anxiety, apathy, and sleep (dependent variables).

Additionally, clinical outcome measures were modeled with cerebral WM FA as the predictor variable. In order to account for potential effects of FSIQ on the relationship between WM FA and clinical outcomes, FSIQ was included if the coefficient was statistically significant.

Finally, a set of models determined the effect of disease duration (predictor variable) on the clinical measures of depression, anxiety, apathy, sleep, and cerebral WM FA (dependent variables).

## Results

Table 2 displays the comparison of the clinical measures and cerebral WM FA across groups. As expected, patients with DM1 had significantly different clinical measures compared to healthy adults. These results included lower FSIQ [*t*_(106)_ = −6.16, *P* < 0.0001], and higher ratings of depression [*t*_(106)_ = 8.12, *P* < 0.0001] and anxiety [*t*_(106)_ = 5.29, *P* < 0.0001). Self-reported apathy [*t*_(106)_ = 5.86, *P* < 0.0001] and informant-reported apathy scores [*t*_(27)_ = 2.43, *P* = 0.0221] were also significantly higher. Additionally, DM1 patients had higher daytime sleepiness scores [*t*_(106)_ = 8.41, *P* < 0.0001]. Finally, cerebral WM FA was significantly lower in the DM1 group compared to healthy adults [*t*_(98)_ = −12.23, *P* < 0.0001].

**Table 2:** Clinical outcome comparisons between groups^a^.

**Variable**	**Estimate**	**95% CI**	***t*-value (df)**	***P*-value**	**R^**2**^**	**R^**2**^ 95% CI**
FSIQ	−14.83	(−19.60, −10.05)	−6.13 (106)	**<0.0001**	0.264	(0.139, 0.402)
Beck depression inventory	7.07	(5.35, 8.80)	8.12 (106)	**<0.0001**	0.384	(0.255, 0.513)
Beck anxiety inventory	5.48	(3.43, 7.53)	5.29 (106)	**<0.0001**	0.209	(0.093, 0.347)
AES: self-report	7.03	(4.65, 9.41)	5.86 (106)	**<0.0001**	0.245	(0.122, 0.383)
AES: informant-report	10.32	(1.60, 19.04)	2.43 (106)	**0.0221**	0.225	(0.026, 0.504)
SCOPA: hypersomnolence	3.90	(2.98, 4.82)	8.41 (106)	**<0.0001**	0.400	(0.271, 0.527)
Cerebral WM FA	−0.05	(−0.05,−0.04)	−12.23 (106)	**<0.0001**	0.604	(0.497, 0.700)

a *All regression coefficients (estimate), 95% confidence intervals, t-values, P-values, and semi-partial R^2^ were calculated in the linear regression model, using group, age, and sex as covariates*.

*AES, apathy evaluation scale; SCOPA, scales for outcomes in Parkinson's disease; FSIQ, full scale intelligence quotient*.

*Bold values indicate they are significant at a p-value < 0.05*.

BDI-II depression scores were subdivided into ranges, where 99% of healthy adults scored in the normal or minimal depression range, and 1% in the mild depression range. For participants with DM1, 72% were in the normal or minimal range, 18% were in the mild range, 10% scored in the moderate range, and none scored in the severe range.

Table 3 shows the results of the models that determined associations between FSIQ and other clinical measures. FSIQ was significantly associated with depression scores [*t*_(35)_ = −3.60, *P* < 0.001] and anxiety scores [*t*_(35)_ = −2.47, *P* = 0.018], with lower FSIQ associated with higher scores of both scales. However, FSIQ was not associated with apathy (neither self-reported or informant-reported scores) or daytime sleepiness.

**Table 3:** FSIQ as a predictor of clinical outcomes in DM1^a^.

**Variable**	**β**	**β 95% CI**	***t*-value (df)**	***P*-value**	**R^**2**^**	**R^**2**^ 95% CI**
Beck depression inventory	−0.50	(−0.78, −0.22)	−3.60 (35)	**<0.001**	0.270	(0.069, 0.510)
Beck anxiety inventory	−0.38	(−0.70, −0.07)	−2.47 (35)	**0.018**	0.149	(0.008, 0.392)
AES: self-report	−0.28	(−0.58, 0.01)	−1.96 (35)	0.058	0.099	(0.001, 0.332)
AES: informant-report	0.08	(−0.31, 0.46)	0.43 (21)	0.675	0.009	(0.000, 0.245)
SCOPA: hypersomnolence	−0.18	(−0.51, 0.14)	−1.13 (35)	0.266	0.035	(0.000, 0.232)

a *All standardized regression coefficients (β), 95% confidence intervals, t-values, P-values, and semi-partial R^2^ were calculated in the linear regression model, using group, age, and sex as covariates*.

*AES, Apathy evaluation scale; SCOPA, scales for outcomes in Parkinson's disease; FSIQ, full scale intelligence quotient*.

*Bold values indicate they are significant at a p-value < 0.05*.

Table 4 shows the results of the analyses evaluating associations between cerebral WM FA and clinical measures. Cerebral FA was strongly associated with FSIQ, where higher FA was associated with higher FSIQ [*t*_(29)_ = 3.61, *P* = 0.001]. FSIQ was included in the models predicting depression and anxiety with FA. There was no statistically significant relationship between FA and depression [*t*_(29)_ = −0.49, *P* = 0.679] or anxiety [*t*_(29)_ = 0.35, *P* = 0.731]. Cerebral FA was not associated with apathy self-reported scores [*t*_(29)_ = −1.88, *P* = 0.071], but was significantly associated with apathy informant-reported scores with small effect size [*t*_(29)_ = ]2.20, *P* = 0.042]. Greater white matter abnormality (lower FA) was associated with higher informant-reported apathy scores. Finally, FA was also significantly associated with hypersomnolence with small effect size [*t*_(29)_ = −2.22, *P* = 0.034], with greater white matter abnormality being associated with higher sleepiness scores.

**Table 4:** Cerebral white matter FA as a predictor of clinical outcomes in DM1^a^.

**Variable**	**β**	**β 95% CI**	***t*-value (df)**	***P*-value**	**R^**2**^**	**R^**2**^ 95% CI**
FSIQ	0.55	(0.24, 0.86)	3.61 (29)	**0.001**	0.309	(0.083, 0.566)
Beck Depression Inventory^*^	−0.08	(−0.50, 0.33)	−0.49 (29)	0.679	0.006	(0.000, 0.189)
Beck Anxiety Inventory^*^	0.07	(−0.36, 0.51)	0.35 (29)	0.731	0.004	(0.000, 0.183)
AES: self-report	−0.30	(−0.64, 0.03)	−1.88 (29)	0.071	0.108	(0.001, 0.370)
AES: informant-report	−0.41	(−0.81, −0.02)	−2.20 (19)	**0.042**	0.222	(0.006, 0.572)
SCOPA: hypersomnolence	−0.37	(−0.71, −0.03)	−2.22 (29)	**0.034**	0.145	(0.004, 0.414)

a *All standardized regression coefficients (β), 95% confidence intervals, t-values, P-values, and semi-partial R^2^ were calculated in the linear regression model, using group, age, and sex as covariates*.

*AES, apathy evaluation scale; SCOPA, scales for outcomes in Parkinson's disease; FSIQ, full scale intelligence quotient*.

* *FSIQ was entered into the model as a covariate*.

*Bold values indicate they are significant at a p-value < 0.05*.

**Figure 1:**
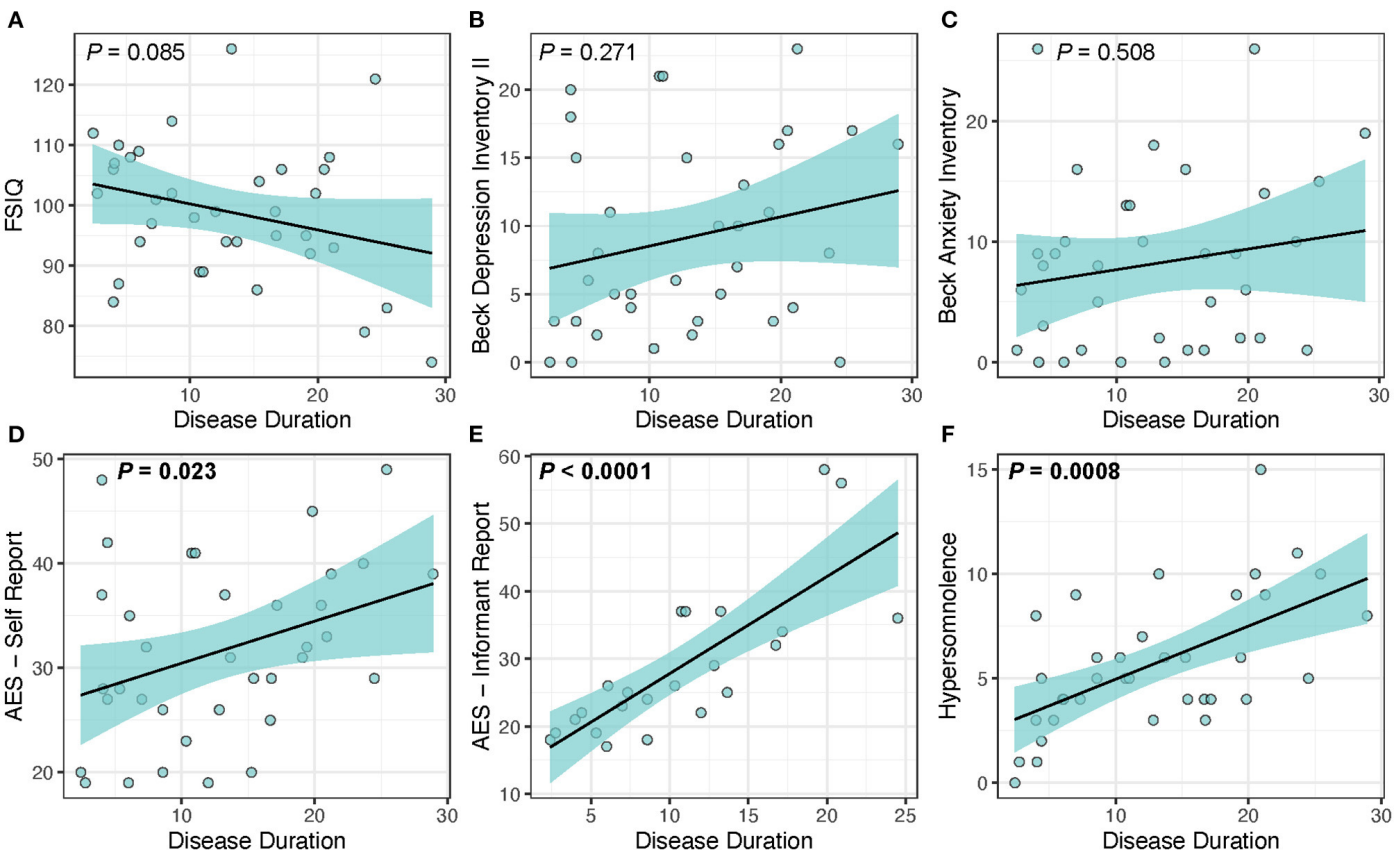
Disease duration as a predictor of clinical measures in DM1. Disease duration was not associated with changes in **(A)** FSIQ (*P* = 0.085), **(B)** depression (*P* = 0.271), or **(C)** anxiety (*P* = 0.508), but was significantly associated with increased scores for the core symptoms of **(D**) self-reported apathy (*P* = 0.023), **(E)** informant reported-apathy (*P* < 0.0001), and **(F)** hypersomnolence (*P* = 0.0008). Light-blue shaded region represents 95% confidence interval.

We found that disease duration (used as a measure of disease progression) was significantly associated with cerebral FA [*t*_(32)_ = −4.28, *P* < 0.001] supporting the hypothesis that white matter integrity decreases over time. Table 5 and Figure 1 shows the results of the analysis evaluating associations between disease duration with clinical measures and cerebral WM FA. We found that disease duration (used as a marker for disease progression) was significantly associated with apathy with a large effect size [*t*_(32)_ = 6.33, *P* < 0.0001]. Disease duration was also significantly associated with hypersomnolence with moderate effect size [*t*_(32)_ = 3.69, *P* < 0.001]. Disease duration was not associated with intelligence quotient [*t*_(32)_ = −1.78, *P* = 0.085], depression [*t*_(32)_ = 1.12, *P* = 0.271], or anxiety [*t*_(32)_ = 0.67, *P* = 0.508] (Figure 1).

**Table 5:** Disease duration as a predictor of clinical outcomes in DM1^a^.

**Variable**	**β**	**β 95% CI**	***t*-value (df)**	***P*-value**	**R^**2**^**	**R^**2**^ 95% CI**
FSIQ	−0.31	(−0.67, 0.05)	−1.78 (32)	0.085	0.090	(0.001, 0.332)
Beck depression inventory	0.19	(−0.16, 0.55)	1.12 (32)	0.271	0.038	(0.000, 0.153)
Beck anxiety inventory	0.12	(−0.24, 0.48)	0.67 (32)	0.508	0.014	(0.000, 0.251)
AES: self-report	0.37	(0.05, 0.69)	2.38 (32)	**0.023**	0.150	(0.010, 0.421)
AES: informant-report	0.72	(0.48, 0.96)	6.33 (19)	**<0.0001**	0.678	(0.460, 0.843)
SCOPA: hypersomnolence	0.54	(0.24, 0.84)	3.692 (32)	**0.0008**	0.299	(0.083, 0.546)
Cerebral FA	−0.68	(−1.00, −0.35)	−4.277 (32)	**0.0002**	0.413	(0.162, 0.656)

a *All standardized regression coefficients (β), 95% confidence intervals, t-values, P-values, and semi-partial R^2^ were calculated in the linear regression model, using group, age, and sex as covariates*.

*AES, apathy evaluation scale; SCOPA, scales for outcomes in Parkinson's disease; FSIQ, full scale intelligence quotient*.

*Bold values indicate they are significant at a p-value < 0.05*.

## Discussion

Brain involvement in DM1 has increasingly become a focus of attention over the past decade, in part because many of the symptoms of DM1 that are attributable to the brain can be quite distressing to patients and their families (43, 64, 65). However, in the context of understanding the neurobiology of DM1 and moving toward reliable therapeutic targets, it is vital to be able to identify which symptoms are related to brain pathology and which ones are secondary to the stress of dealing with a chronic disease and not due to underlying brain pathology (15).

Previous studies have shown significant relationships between neuroimaging findings and cognitive impairments, including white matter track DTI measures, ventricular enlargement, gray matter and white matter atrophy, white matter lesions, increased hippocampal volume, and FDG-PET frontotemporal hypometabolism (4, 9, 12, 19, 66–68). Previous studies have also confirmed cognitive decline over time with age and a negative association with disease duration (49, 50, 69, 70).

In support of the hypothesis that depression and anxiety are not a manifestation of DM1 brain pathology, there was no relationship to the primary brain imaging abnormality in DM1 of decreased white matter FA. In addition, Hamilton et al. (71) found no correlation between white matter lesion volume (mL) and BDI-II scores and Winblad et al. (27) found that white matter lesions may actually protect against depression. In addition, Winblad et al. (27) found that depression was associated with earlier stages of DM1 and was negatively correlated to disease progression (27). The construct of depression as a clinical disorder is predicated on a foundational principle of an altered and depressed mood (i.e., feeling “sad”). A previous study by Serra et al. (72) showed significant associations between cortical thickness and social cognition performance, including sadness. However, this study did not include any depression-specific scales and had a relatively small sample size. In our current sample, the DM1 patients, despite scoring high on other BDI-II items, did not report feeling sad (Mean DM1 score on this item was 0.078, mean healthy adult score was 0.028). Moreover, although individuals with DM1 scored higher on the BDI-II, the healthy adult group in our study was not the optimal comparison group. That is, they were healthy and not suffering from a chronic, degenerative medical condition. This suggests that the BDI-II is reflecting the physical symptoms of DM1 rather than the emotional symptoms that are more commonly associated with depression. This is supported by the fact that there are no differences in the BDI-II affective and somatic sub-domains in our study. Winblad et al. (27) also found that DM1 patients tend to have high scores on somatic items and low scores on cognitive-affective items of the BDI-II. In summary, these findings support the hypothesis that what is likely being measured by the BDI-II (or other standard depression rating scales) in DM1 is a combination of physical symptoms of their disease and any emotional/affective features that are not part of a true mood disorder. These symptoms are instead the manifestation of the stress involved in dealing with a chronic and debilitating neurological condition. Our findings also highlight the concept that these affective symptoms associated with a chronic disease are heightened in those with a cognitive disorder as lower FSIQ was associated with higher depression and anxiety ratings. It has also been shown that increased cognitive impairment is significantly correlated with higher BDI-II scores (71). It is likely that DM1 patients with cognitive deficits are using more emotion-focused coping strategies instead of problem-focused coping strategies (73). In summary, we conclude that depression (or anxiety) is not a core feature of DM1 (27, 74).

Apathy, a disorder of diminished motivation, has long been associated with DM1, with a prevalence of 40–55% (42). Apathy has been associated with impairments in social, occupational, and functional domains including impairments in activities of daily living, diminished quality of life, increased caregiver burden, and treatment non-adherence (17). An interesting core feature of apathy is that it is often not bothersome to the patient and sometimes not even acknowledged as problematic (lack of insight). In contrast, family members may express extreme frustration with their loved-one's apathy. Although in our sample, there was wide variance in agreement between a patient's self-rated apathy score and the informant-rated apathy score (ranging from the patient scoring 20 points greater than the informant to the informant scoring 23 points greater than the patient), it was the informant ratings that had the strongest relationship with brain FA with a larger effect size. This suggests that the informant-rated scores may be a more accurate and consistent indicator of brain pathology. Additionally, Guercio et al. (75) found that self-reported apathy scores in Alzheimer's disease are less reliable if the subjects have cognitive impairment.

Finally, daytime sleepiness or hypersomnolence is a core feature of DM1 brain pathology with the current findings showing strong relationships to low FA. Previous studies have also shown significant relationships between brain pathology and neuroimaging findings with sleep problems, including ventricular enlargement, gray and white matter atrophy, white matter lesions, decreased volume of the pallidum and diencephalon, hypoechogenic raphe, iron accumulation in the caudate nucleus, and decreased neurofibrillary tangles (6, 22, 26, 76, 77). Moreover, white matter tract mean diffusivity in the superior longitudinal fasciculus and cingulum has been associated with an increased score on the Epworth Sleepiness Scale (4). The prevalent symptom of hypersomnolence causes major morbidity in DM1 (16, 45, 78–81). Although relatively crudely tracked by self-report in this current study, this feature can also be evaluated by quantitative assessment such as EEG from polysomnography, which could be used in future studies or clinical trials (82).

A limitation of this present study is a relatively low median CTG repeat length of 146 and mild MIRS score compared to other studies, suggesting that the DM1 group is only mildly affected. However, our study was primarily focused on adult-onset DM1, and by excluding congenital, childhood, and juvenile forms of DM1 the median CTG repeat length of 146 was not unexpected. This could lead to problems generalizing these results to a larger DM1 population that included congenital, childhood, and juvenile DM1 patients. Like most human research studies, adult-onset DM1 participants that are moderately to severely affected face greater barriers with research study participation, thus biasing the study population itself to those less affected by disease. Another limitation to this study is the use of self-report questionnaires for measurement of depression, anxiety, apathy, and hypersomnolence. This could have led to biased results through misreporting of symptoms.

The current findings highlight the concept that core features of the disease that *are* directly related to measures of brain pathology (lower WM FA) are FSIQ, apathy, and hypersomnolence. Additionally, as a measure of disease progression, disease duration was significantly associated with the core features of apathy, hypersomnolence, and cerebral FA. Identifying features that represent the true manifestation of brain pathology in DM1 is of particular importance given that drug companies are interested in targeting the CNS for clinical trials of gene knock-down and other therapies. It is important to note that a correlation to disease duration is only a proxy to disease progression. Moreover, given the wide range of disease duration in our sample, it will be possible to evaluate disease progression in the future. Whether progression in these symptoms occur rapidly is important to assess in the context of whether or not they may represent appropriate end-points for clinical trials. Our study is designed as a prospective longitudinal study and evaluation of change over a one- and two-year time period is currently underway.

## Conclusions

Our study supports the hypothesis that the underlying neuropathology of adult-onset myotonic dystrophy type 1, as measured by cerebral WM FA and disease duration, leads directly to cognitive deficits, apathy, and hypersomnolence, while increased symptoms of depression and anxiety are secondary to a combination of the physical symptoms of having a neuromuscular disorder and the emotional stress of coping with a chronic and debilitating disorder.

## Data Availability Statement

The raw data supporting the conclusions of this article will be made available by the authors, without undue reservation.

## Ethics Statement

The studies involving human participants were reviewed and approved by University of Iowa Institutional Review Board. The patients/participants provided their written informed consent to participate in this study.

## Author Contributions

Conceptualization and Methodology, Formal Analysis, Writing—Original Draft: JM and PN. Investigation: JM, PN, AK, DJM, LG, EP, TK, SC, and DGM. Resources, Supervision, and Funding Acquisition: PN. Writing—Review & Editing: JM, PN, AK, DJM, LG, EP, TK, SC, and DGM. Visualization: JM. All authors had full access to all the data in the study and take responsibility for the integrity of the data and the accuracy of the data analysis.

## Conflict of Interest

Within the last three years DM has been a scientific consultant and/or received an honoraria/stock options from AMO Pharma, Charles River, Vertex Pharmaceuticals, Triplet Therapeutics, LoQus23 and Small Molecule RNA. DM also had/has research contracts with AMO Pharma and Vertex Pharmaceuticals. The remaining authors declare that the research was conducted in the absence of any commercial or financial relationships that could be construed as a potential conflict of interest.

## Abbreviations

AES: apathy evaluation scale
BDI-II: beck depression inventory-II
BDA: beck anxiety inventory
DM1: myotonic dystrophy type 1
DTI: diffusion tensor imaging
EEG: electroencephalography
ePAL: estimated progenitor allele length
FA: fractional anisotropy
MRI: magnetic resonance imaging
FSIQ: full scale intelligence quotient
SCOPA: scales for outcomes in Parkinson's disease
SP-PCR: small-pool polymerase chain reaction
WAIS-IV: Wechsler adult intelligence scale-IV
WM: white matter.
